# Contrast-Enhanced Endoscopic Ultrasonography for Pancreatic Tumors

**DOI:** 10.1155/2015/491782

**Published:** 2015-05-18

**Authors:** Yasunobu Yamashita, Jun Kato, Kazuki Ueda, Yasushi Nakamura, Yuki Kawaji, Hiroko Abe, Junya Nuta, Takashi Tamura, Masahiro Itonaga, Takeichi Yoshida, Hiroki Maeda, Takao Maekita, Mikitaka Iguchi, Hideyuki Tamai, Masao Ichinose

**Affiliations:** ^1^Second Department of Internal Medicine, Wakayama Medical University, 811-1 Kimiidera, Wakayama City, Wakayama 641-0012, Japan; ^2^Division of Pathology, Department of Clinical Laboratory Medicine, Wakayama Medical University, 811-1 Kimiidera, Wakayama City, Wakayama 641-0012, Japan

## Abstract

*Objectives*. To investigate the usefulness of contrast-enhanced endoscopic ultrasonography (CE-EUS) for histological differentiation of pancreatic tumors. *Methods*. CE-EUS was performed for consecutive patients having a pancreatic solid lesion, and tumors were classified into three vascular patterns (hypervascular, isovascular, and hypovascular) at two time phases (early-phase and late-phase). Correlation between vascular patterns and histopathology of resected pancreatic cancer (PC) tissues was ascertained. *Results*. The final diagnoses of 147 examined tumors were PC (*n* = 109), inflammatory mass (*n* = 11), autoimmune pancreatitis (*n* = 9), neuroendocrine tumor (*n* = 8), and others (*n* = 10). In late-phase images, 104 of 109 PCs had the hypovascular pattern, for a diagnostic sensitivity and specificity of 94% and 71%, respectively. Of 28 resected PCs, 10 had isovascular, and 18 hypovascular, patterns on the early-phase image. Early-phase isovascular PCs were more likely to be differentiated than were early-phase hypovascular PCs (6 well and 4 moderately differentiated versus 3 well, 14 moderately, and 1 poorly differentiated, *P* = 0.028). Immunostaining revealed that hypovascular areas of early-phase images reflected heterogeneous tumor cells with fibrous tissue, necrosis, and few vessels. *Conclusion*. CE-EUS could be useful for distinguishing PC from other solid pancreatic lesions and for histological differentiation of PCs.

## 1. Introduction

Pancreatic cancer (PC) carries dismal prognosis with a 5-year overall survival rate of 6% [1]. The incidence of PC has increased steadily over the last four decades; it is the fourth leading cause of cancer mortality in the USA and was responsible for an estimated 37,390 deaths in 2012 [1]. Even with recent advances in diagnostic imaging technology, most cases of PC are only discovered at an unresectable stage, when prognosis is poor and the 5-year survival rate is 2% [1]. Endoscopic ultrasonography (EUS) is thought to be one of the most reliable and efficient diagnostic modalities for pancreatobiliary diseases because of its superiority to any other modalities with respect to spatial resolution [2, 3]. Despite its ability to detect small pancreatic lesions with high sensitivity, EUS alone has limitations in distinguishing PC from nonneoplastic pancreatic masses, because most pancreatic solid lesions are depicted as a hypoechoic mass regardless of histology [4].

To date, diagnosis of PC has been made with tissues obtained by EUS-guided fine-needle aspiration (FNA). However, there are cases where the diagnosis is still difficult with EUS-FNA because the aspirate contains insufficient tumor material; and there are contraindications to EUS-FNA, such as concurrent coagulopathy. Moreover, EUS-FNA poses the risk of seeding tumor cells and bleeding. Therefore, development of alternatives to FNA as a modality for differentiating PC from other pathological lesions is eagerly anticipated.

Contrast-enhanced-EUS (CE-EUS) detects signals from microbubbles produced by intravenously administered contrast agents and filters signals originating from tissues, by selectively detecting harmonic components. This technology can detect signals from microbubbles in vessels having very slow flow without Doppler-related artifacts and is used to characterize vascularity [5, 6]. Kitano et al. reported the largest series of 277 patients with a solid pancreatic lesion who underwent CE-EUS. CE-EUS-depicted hypoenhancement with Sonazoid (Daiichi Sankyo Co. Ltd., Tokyo, Japan) diagnosed PC with a sensitivity and specificity of 95% and 89%, respectively. When compared with multidetector contrast-enhanced computed tomography (CT), CE-EUS yielded a significantly higher accuracy in diagnosing pancreatic adenocarcinomas that were less than 2 cm in size, with a sensitivity and specificity of 91% and 94%, respectively [7].

We previously reported that tumor vascularity visualized on CE-EUS enabled differentiation of mural nodules from mucous clots in intraductal papillary mucinous neoplasms of the pancreas [8] and prediction of chemotherapy efficacy in PC patients [9]. Thus, CE-EUS can noninvasively evaluate pancreatic tumors to some extent. However, neither CE-EUS findings for tumors other than PC nor the correlation between images of CE-EUS and histology of PCs has been fully evaluated, to our knowledge, although contrast-enhanced ultrasonography (CE-US) has been used for estimation of histological differentiation of hepatocellular carcinoma [10].

In this study, therefore, CE-EUS was performed for consecutive patients with a pancreatic solid lesion, and CE-EUS findings for PC and other pathological lesions were examined. Moreover, CE-EUS images were compared with histology findings, including immunostaining, of PCs after surgical resection.

## 2. Materials and Methods

### 2.1. Patients

Between February 2009 and July 2013, we performed B-mode EUS and CE-EUS for consecutive patients having a pancreatic solid lesion found on CT or magnetic resonance imaging (MRI). Tumors with a cystic component greater than 50% of the total lesion volume were not eligible for enrollment in this study. In total, 147 patients underwent the study procedures during the designated period. The final diagnoses were determined by histopathological examination of specimens obtained by surgery, EUS-FNA, or biopsy of liver metastases. The diagnosis of autoimmune pancreatitis (AIP) was based on the international consensus diagnostic criteria for AIP [11]. Histology of PC of the patients who underwent surgical resection was classified as well-, moderately, or poorly differentiated adenocarcinoma according to World Health organization criteria [12]. All histological evaluations were performed by the same board-certified pathologist.

This study was approved by the ethics committee of Wakayama Medical University and informed consent was obtained from each patient.

### 2.2. EUS Procedure

Electronic radial-type and convex-type endoscopes (GF-UE260-AL5, GF-UCT260; Olympus, Tokyo, Japan) with ultrasound observation systems (ALOKA ProSound SSD *α*-10; Aloka Co. Ltd, Tokyo, Japan) were used. The ultrasound system employed the extended pure harmonic detection method with the mechanical index set at 0.35. EUS was performed in the left lateral position under diazepam-induced sedation with heart rate monitoring.

To perform CE-EUS, Sonazoid, which is a second-generation ultrasonography contrast agent composed of perfluorobutane microbubbles with a median diameter of 2-3 *μ*m [13], was used. After reconstitution with 2 mL of sterile water for injection, 0.7 mL of the agent was administered through a peripheral vein. The underlying principles of contrast harmonic imaging are as follows: when exposed to ultrasound beams, microbubbles in the contrast agent are disrupted or resonate and release many harmonic signals [14]. When tissue and microbubbles receive the transmitted ultrasound waves, both produce harmonic components that are integer multiples of the fundamental frequency; the harmonic components from the microbubbles are higher than those from the tissue. Selective depiction of the second harmonic component visualizes signals from microbubbles more strongly than those from tissue [15].

**Figure 1:**
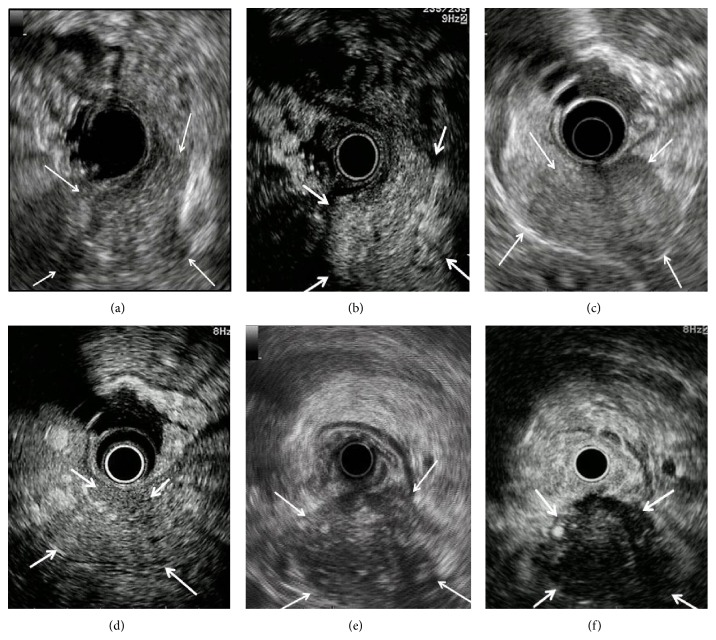
Evaluation of vascular pattern by contrast-enhanced endoscopic ultrasonography (CE-EUS). A representative case of tumors with hypervascular pattern: pancreatic lesion was detected as a low echoic lesion (arrow) with B-mode EUS (a). CE-EUS detected a pancreatic lesion with hyperintensity of enhancement (arrow) as compared to that of surrounding pancreatic tissue (b). A representative case of tumors with isovascular pattern: pancreatic lesion was detected as a low echoic lesion (arrow) with B-mode EUS (c). CE-EUS detected a pancreatic lesion with isointensity of enhancement (arrow) as compared to that of surrounding pancreatic tissue (d). A representative case of tumors with hypovascular pattern: pancreatic lesion was detected as a low echoic lesion (arrow) with B-mode EUS (e). CE-EUS detected a pancreatic lesion with hypo-intensity of enhancement (arrow) as compared to that of surrounding pancreatic tissue (f).

After a bolus infusion of the contrast agent was administered, vascular patterns were continuously monitored in real time. Vascular patterns at two time phases were assessed: 0–15 seconds (early-phase images) and 30–60 seconds (late-phase images) after the injection. All clips were stored in the hard disk of the scanner for offline investigation. Vascular patterns of tumors, as contrasted with those of the surrounding pancreatic tissue, were determined and classified as hypovascular, isovascular, or hypervascular, for both early-phase and late-phase images (Figure 1). The evaluations were made on-site by two physicians, each having at least 5 years of EUS experience.

### 2.3. Histological Analysis

Histopathological analysis of resected PCs was performed by staining tissue sections with H & E, elastica van Gieson, and Masson trichrome. Immunohistochemical staining by the Envision polymer method was performed to detect cytokeratin using AE1/AE3 (mouse monoclonal, prediluted; Nichirei, Tokyo, Japan) as the primary antibody and the autoimmunostainer histostainer 48A (Nichirei, Tokyo, Japan), according to the manufacturer's instructions.

### 2.4. Statistical Analysis

The McNemar test was used to evaluate the correlation between vascular pattern as determined by CE-EUS and the extent of histopathological differentiation. A difference was considered significant when the *P* value was less than 0.05. Statistical analysis was performed with SPSS software (version 11) (SPSS, Chicago, IL, USA).

## 3. Results

A total of 147 patients with a solid pancreatic lesion were examined (92 males, 55 females; median age 69 years, range 32–91 years). Average lesion size was 30 mm (range, 8–107 mm). The final diagnoses of the lesions were as follows: PC (*n* = 109); acinar cell carcinoma (*n* = 2); inflammatory mass (*n* = 11); neuroendocrine tumor (NET) (*n* = 8); AIP (*n* = 9); invasive intraductal papillary mucinous carcinoma (IPMC) (*n* = 5); metastatic lesions (*n* = 2: lung cancer 1, melanoma 1); and intraductal tubular tumor (ITT) (*n* = 1) (Table 1).

The echo images on B-mode EUS were isoechoic (*n* = 6) or hypoechoic (*n* = 141) (Table 2). The vascular patterns of CE-EUS early-phase and late-phase images for 147 pancreatic solid lesions are also shown in Table 2. With regard to late-phase images, 102 of 109 (94%) PCs had the hypovascular pattern, compared to 11 of the remaining 38 (29%) non-PC lesions. The hypervascular pattern was observed in NET tumors only (5/8, 63%) (Table 2). Detection of the hypovascular pattern in the CE-EUS late-phase image diagnosed PC with sensitivity, specificity, and accuracy of 94%, 71%, and 88%, respectively.

Most non-PC tumors did not have differences in the vascular pattern between the early-phase and late-phase images (patterns unchanged: 34/38, 89%). In contrast, although the majority of PCs had the hypovascular pattern in late-phase images, early-phase images of PCs were clearly divided into two groups: 40 had the isovascular pattern with homogeneous enhancement, and 69 had the hypovascular pattern with heterogeneous enhancement. The histological differentiation of 28 surgically resected PCs was evaluated in comparison with their early-phase images. Of the 10 PCs having isovascular early-phase images, 6 (60%) were well-differentiated tubular adenocarcinoma and 4 (40%) were moderately differentiated tubular adenocarcinoma. In contrast, 18 PCs having hypovascular early-phase images included 3 (17%) well-differentiated adenocarcinomas, 14 (78%) moderately differentiated adenocarcinomas, and 1 poorly differentiated adenocarcinoma. The degree of vascularity detected on the CE-EUS early-phase image was significantly correlated with the histological differentiation of PCs (*P* = 0.028) (Table 3).

**Figure 2:**
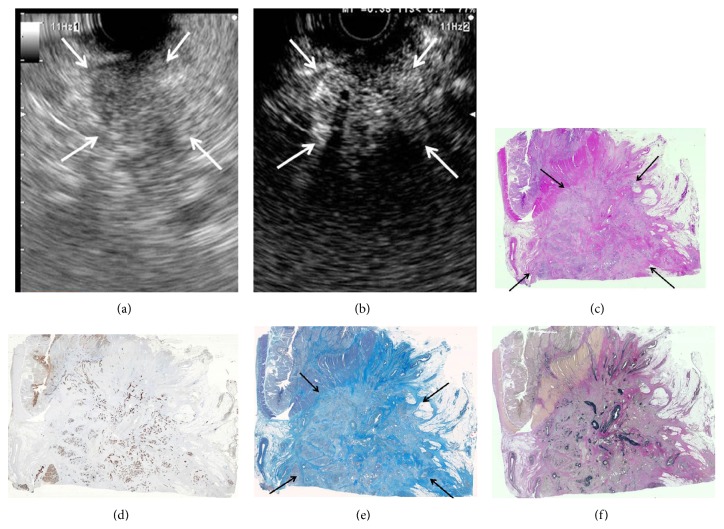
Correlation of early-phase CE-EUS findings (b) and staining data in serial sections of resected pancreatic cancer tissue (c–f): a case of the isovascular tumor. (a) Pancreatic lesion was detected as a low echoic lesion (arrow) with B-mode EUS. (b) Early-phase CE-EUS image reveals isovascular tumor (arrow) with homogeneous enhancement. (c) Resected tumor specimen (arrow) without necrotic tissue: hematoxylin-eosin staining (original magnification ×1). (d) Cytokeratin staining revealed that the isovascular area on CE-EUS was composed of a homogeneous population of tumor cells (original magnification ×1). (e) Masson trichrome staining showed no fibrous tissues in the area (arrow) (original magnification ×1). (f) Homogeneous area of abundant vessels was observed on elastica van Gieson staining (original magnification ×1).

**Figure 3:**
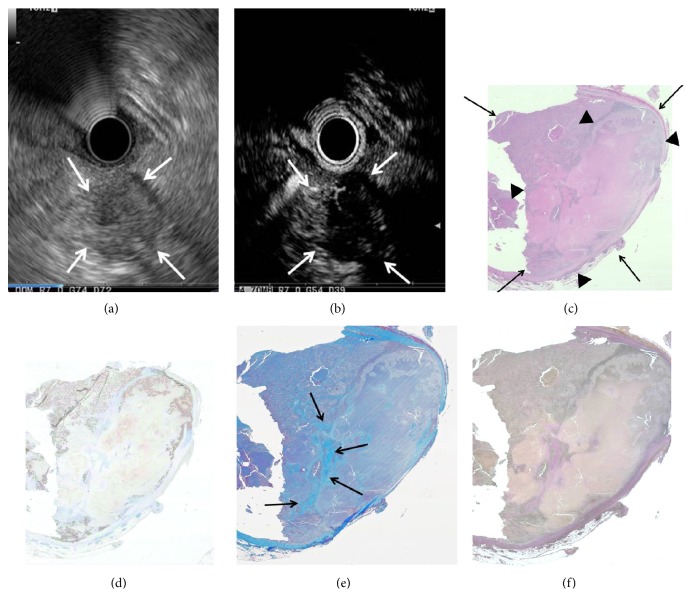
Correlation of early-phase CE-EUS findings (b) and staining data of serial sections of resected pancreatic cancer tissue (c–f): a case of the hypovascular tumor. (a) Pancreatic lesion was detected as a low echoic lesion (arrow) with B-mode EUS. (b) CE-EUS revealed hypovascular tumor (arrow) with heterogeneous enhancement in early-phase image. (c) Resected tumor specimen (arrow) with necrotic area (arrowhead): hematoxylin-eosin staining (original magnification ×1). (d) Cytokeratin staining revealed heterogeneous tumor cells (original magnification ×1). (e) Masson trichrome staining disclosed the presence of fibrous tissues (arrow) (original magnification ×1). (f) Few vessels were observed on elastica van Gieson staining (original magnification ×1).

We next assessed what histology was reflected by the isovascular and hypovascular areas on early-phase images by immunostaining of tissue sections of the resected PCs. Elastica van Gieson, Masson trichrome, and cytokeratin staining revealed that isovascular areas in early-phase images correlated with areas of homogeneous tumor cells with abundant vessels and without fibrous tissues (Figure 2); on the other hand, hypovascular areas reflected areas of heterogeneous tumor cells with fibrous tissue, necrosis, and few vessels (Figure 3).

## 4. Discussion

In this study, we performed CE-EUS for consecutive patients with a solid pancreatic tumor. Because a large portion of pancreatic tumors detected on CT or MRI were PC, our data are mostly from PC, similar to previous reports [4, 7, 16, 17]. Although the usefulness of CE-EUS for diagnosis of PC has been reported previously, the current study had several strong points. First, several kinds of tumors (or pseudotumors) other than PC were examined, and characteristics of CE-EUS images of those relatively rare, but clinically important, tumors were shown. Next, we analyzed CE-EUS images at two time points to obtain early-phase and late-phase images. Previous reports analyzed late-phase images alone, and the information has been limited. Addition of analysis of early-phase, and the combination of early-phase and late-phase, images revealed novel aspects of the potential usefulness of CE-EUS for pancreatic tumor patients. Lastly, correlation of CE-EUS images with histopathology of resected PC specimens was found. Analysis of early-phase images enabled the clinically relevant comparison between CE-EUS images and PC histology.

CE-EUS images of the majority of the diagnosed PCs were found to have a hypovascular pattern and lower intensity of enhancement relative to the surrounding pancreatic tissue. Yamashita et al. reported that CE-EUS-depicted hypoenhancement with Sonazoid diagnosed PC with a sensitivity and specificity of 95% and 89%, respectively [8]. Three other groups [4, 16, 17] reported that CE-EUS with SonoVue (sulfur hexafluoride MBs, Bracco, Italy) could be used to diagnose PCs with high sensitivity (93%, 89%, and 96%, resp.). SonoVue and Sonazoid are the most commonly used contrast agents in recent studies. The two ultrasound contrast agents differ in terms of intensities and durations of signaling after infusion. CE-EUS images obtained with SonoVue disappear within 60 seconds, which limits the duration of observation. In contrast, after infusion of Sonazoid, late-phase can be observed throughout the pancreas for at least 90 seconds. Thus, the longer-lasting effect of Sonazoid improves the observation of the pancreas by CE-EUS [8]. In our study with Sonazoid, the sensitivity and specificity of CE-EUS for PC were 94% and 71%, respectively, which were equivalent to the values of the previous report. Moreover, PCs having the isovascular pattern in early-phase images were likely to develop the hypovascular pattern in the late-phase, while non-PC tumors having the isovascular early-phase pattern were not likely to change during the late-phase (image changed, 83% versus 13%, *P* < 0.01). This information could help clinicians narrow the differential diagnosis of pancreatic lesions.

As for CE-EUS images of pancreatic tumors (or pseudotumors) other than PC, Kitano et al. reported that 36 of 46 (78%) of inflammatory masses had the isovascular pattern, while 15 of 19 (78%) NETs had the hypervascular pattern [7]. Matsubara et al. defined AIP and inflammatory mass as inflammatory pancreatic pseudotumor (IPPT) and reported that 21 of 27 (78%) IPPTs (AIP, 14; inflammatory mass, 13) had the isovascular pattern [18]. In our study, similar results were obtained: 8 of 11 (72%) inflammatory masses and 8 of 9 (89%) AIPs were isovascular; and 5 of 8 (63%) NETs were hypervascular. To our knowledge, this is the first study to demonstrate CE-EUS vascular patterns for acinar cell carcinoma and ITT. However, because the numbers of tumors examined were small, further studies are required to confirm these results. As mentioned above, the unchanged nature of early-phase and late-phase CE-EUS images was one of the characteristics of non-PC tumors in this study.

The highlight of this study was the original finding that PCs clearly divided into isovascular or hypovascular patterns on early-phase CE-EUS images. Moreover, this difference reflected the degree of histological differentiation of the PC. Assessment of histological differentiation impacts clinical care because histologic poorly differentiated adenocarcinoma has been recognized largely as an independent, poor prognostic factor for PC [19]. The long-lasting effect of Sonazoid revealed these new findings. In future studies, the correlation among early-phase CE-EUS images, histological differentiation of tumors, and clinical characteristics of PCs, including chemosensitivity and prognosis, should be elucidated.

Special staining and immunostaining showed what histology CE-EUS early-phase images reflected. The hypovascular pattern corresponded with heterogeneous tumor cells with necrotic tissue, fibrous tissue, and few vessels, while the isovascular pattern corresponded with homogeneous tumor cells with abundant vessels and no necrotic or fibrous tissues. These findings would be relevant for the noninvasive evaluation of biological features of PC as well as non-PC tumors and may develop further indications for use of CE-EUS and the contrast agent in the field other than PC. Moreover, the results may be helpful in current clinical settings involving EUS-FNA assessment. Diagnosing tumors with heterogeneous cells and necrotic tissues by EUS-FNA would be unsuccessful. CE-EUS early-phase images may identify tumors which are difficult to assess by EUS-FNA. In fact, in our experience of 61 EUS-FNA procedures, all 8 cases in which diagnosis was not possible from the obtained sample exhibited the hypovascular pattern.

A major limitation of this study was the small number of resected PCs enrolled. More substantive analyses, such as the correlation between CE-EUS images and clinical outcomes of PC, should be performed using a larger number of surgically resected tumors.

In conclusion, evaluation of vascularity by CE-EUS was useful for differentiating PCs from other solid pancreatic lesions and for the histological evaluation of PCs. More extensive analysis in the future of CE-EUS images may demonstrate that this modality can be an alternative to EUS-FNA for the diagnosis of pancreatic tumors.

## Figures and Tables

**Table 1:** Patient characteristics.

Characteristic	Value
Median age (range), years	69 (32–91)
Sex (male : female)	92 : 55
Median size of lesions (range), mm	30 (8–107)
Final diagnosis	
Pancreatic cancer	109
Acinar cell carcinoma	2
Inflammatory mass	11
Neuroendocrine tumor	8
Autoimmune pancreatitis	9
Invasive intraductal papillary mucinous carcinoma	5
Metastasis	2
Intraductal tubular tumor	1

**Table 2:** Comparison of contrast-enhanced endoscopic ultrasonography and final diagnosis.

	Pancreatic cancer (*n* = 109)	Inflammatory mass (*n* = 11)	NET (*n* = 8)	AIP (*n* = 9)	Invasive IPMC (*n* = 5)	Metastatic lesion (*n* = 2)	ITT (*n* = 1)	Acinar cell carcinoma (*n* = 2)
B-mode EUS								
Hypoechoic	105	11	7	9	5	2	0	2
Isoechoic	4	0	1	0	0	0	1	0
CE-EUS								
Early-phase image								
Hypovascular pattern	69	3	0	1	3	1	0	0
Isovascular pattern	40	8	2	8	2	1	1	2
Hypervascular pattern			6					
Late-phase image								
Hypovascular pattern	102	3	1	1	3	2	1	0
Isovascular pattern	7	8	2	8	2	0	0	2
Hypervascular pattern			5					

NET, neuroendocrine tumor; AIP, autoimmune pancreatitis; IPMC, intraductal papillary mucinous carcinoma; ITT, intraductal tubular tumor; EUS, endoscopic ultrasonography; CE-EUS, contrast-enhanced endoscopic ultrasonography.

**Table 3:** Correlation between vascular pattern on early-phase contrast-enhanced endoscopic ultrasonography image and histological differentiation of pancreatic cancers.

	Isovascular pattern in early- phase	Hypovascular pattern in early-phase
Pathological finding		
Well	6	3
Moderate	4	14
Poor	0	1
		*P* = 0.028

Well, well-differentiated tubular adenocarcinoma; moderate, moderately differentiated tubular adenocarcinoma; poor, poorly differentiated carcinoma.
